# Prostate SBRT using O‐Ring Halcyon Linac — Plan quality, delivery efficiency, and accuracy

**DOI:** 10.1002/acm2.13105

**Published:** 2020-12-19

**Authors:** Damodar Pokhrel, Tanner Tackett, Joseph Stephen, Justin Visak, Falguni Amin‐Zimmerman, Andrew McGregor, Stephen E. Strup, William St Clair

**Affiliations:** ^1^ Medical Physics Graduate Program Department of Radiation Medicine University of Kentucky Lexington KY USA; ^2^ Lexington Clinic University of Kentucky Lexington KY USA; ^3^ Department of Urology University of Kentucky Lexington KY USA

**Keywords:** AcurosXB, Co/non‐planar Geometry, FFF‐beam, Halcyon Linac, Prostate SBRT, VMAT

## Abstract

Cone beam CT‐guided prostate stereotactic body radiotherapy (SBRT) treatment on the recently installed novel O‐ring coplanar geometry Halcyon Linac with a single energy 6MV‐flattening filter free (FFF) beam and volumetric modulated arc therapy (VMAT) is a fast, safe, and feasible treatment modality for early stage low‐ and intermediate‐risk prostate cancer patients. Following the RTOG‐0938 compliance criteria and utilizing two‐full arc geometry, VMAT prostate SBRT plans were generated for ten consecutive patients using advanced Acuros‐based algorithm for heterogeneity corrections with Halcyon couch insert. Halcyon VMAT plans with the stacked and staggered multileaf collimators (MLC) produced highly conformal SBRT dose distributions to the prostate, lower intermediate dose spillage and similar dose to adjacent organs‐at‐risks (OARs) compared to SBRT‐dedicated Truebeam VMAT plans. Due to lower monitor units per fraction and less MLC modulation through the target, the Halcyon VMAT plan can deliver prostate SBRT fractions in and overall treatment time of less than 10 minutes (for 36.25 Gy in five fractions), significantly improving patient compliance and clinic workflow. Pretreatment quality assurance results were similar to Truebeam VMAT plans. We have implemented Halcyon Linac for prostate SBRT treatment in our institution. We recommend that others use Halcyon for prostate SBRT treatments to expand the access of curative hypofractionated treatments to other clinics only equipped with a Halcyon Linac. Clinical follow‐up results for patients who underwent prostate SBRT treatment on our Halcyon Linac is underway.

## 1 INTRODUCTION

Stereotactic body radiation therapy (SBRT) is an established treatment option in the management of early‐stage low‐ or intermediate‐risk prostate cancer patients and provides high cure rates of 90‐100% (median, 3‐4 years, actuarial). These cure rates are possible with <3% of patients suffering from long‐term grade 3 rectal and urinary treatment toxicity.^1^, ^2^, ^3^, ^4^, ^5^, ^6^, ^7^, ^8^, ^9^ Fewer number of prostate SBRT treatments reduce patient hospital/clinic visits and will help to improve patient compliance and clinic efficiency. Traditionally, prostate SBRT treatments have long treatment times and are delivered using a robotic CyberKnife,^1^, ^2^, ^3^, ^4^, ^5^, ^6^, ^7^, ^8^, ^9^ Tomotherapy unit,^10^ or multiple field intensity modulation radiation therapy (IMRT).^11^ The Radiation Therapy Oncology Group (RTOG) 0938 report provides the most recent recommendations for prostate SBRT dosing scheme, contouring, treatment planning, and delivery criteria^12^ in addition to the standard SBRT guidelines.^13^ Due to the relatively longer traditional treatment time and patient discomfort,^1^, ^2^, ^3^, ^4^, ^5^, ^6^, ^7^, ^8^, ^9^, ^10^, ^11^ prostate SBRT has recently been delivered using a SBRT‐dedicated C‐arm Linac via volumetric modulated arc therapy (VMAT).^14^, ^15^, ^16^ VMAT provides a highly conformal dose distribution to the prostate while maintaining fast dose fall‐off outside the target, better sparing of organs‐at‐risk (OAR) and much faster treatment delivery. The dosimetric advantages of VMAT prostate SBRT can be enhanced by utilizing flattening filter free (FFF) beam because of its higher dose rates, reduction in out‐of‐field dose and decreased head scatter and electron contamination with respect to traditional flattened beams.^17^, ^18^ Linac‐based five fractions prostate SBRT treatment have been studied by a few researchers.^19^, ^20^, ^21^ For instance, in a recent phase II study by Alongi et al,^20^ it was demonstrated that five consecutive Linac‐based SBRT treatments of prostate cancer patients to 35 Gy (for low risk) and to 37.5 Gy (for intermediate risk) were well tolerated with comparable oncological outcomes to every other day fractionations. Shortening overall treatment time could potentially reduce intrafraction motion errors and improve daily treatment delivery accuracy therefore improving patient compliance and safety.

A commercially available, fast‐rotating coplanar O‐ring Linac, Halcyon V2.0 (Varian Medical Systems, Palo Alto, CA) was recently introduced by Varian for conventionally fractionated image‐guided radiation therapy (IGRT).^22^ This novel linac was designed under tight performance specifications in order to improve patient safety and treatment accuracy. The Halcyon Linac is equipped with a single‐energy 6MV FFF beam with a rapid gantry rotation speed of four revolutions per minute.^23^, ^24^, ^25^ The Halcyon mean energy and the nominal depth of maximal dose are 1.3 MeV and 1.3 cm, compared to the similar corresponding Truebeam Linac (6MV‐FFF, beam) at 1.4 MeV and 1.5 cm, respectively. In contrast to Truebeam platform, the Halcyon is equipped with a newly designed double stacked and staggered 1 cm width MLC layers. The proximal and distal layers are offset by 5 mm allowing for a projected 5 mm effective MLC width at isocenter, similar to a SBRT‐dedicated Truebeam Linac. The maximal field size of this jawless Halcyon Linac is 28 × 28 cm^2^. The MLC leaves on Halcyon are twice as fast as the standard millennium 120 MLC and their stacked/staggered design allow for very low MLC leakage and transmission of <0.5%.^25^, ^26^ Halcyon provides an improved penumbra with a smaller dosimetric leaf gap (DLG) of 0.1 mm. In addition to MV‐cone beam CT imaging, the Halcyon Linac is equipped with a fast 15‐second kilovoltage cone beam CT (kV‐CBCT) imaging system that includes a high‐quality iterative CBCT reconstruction algorithm (iCBCT).^27^, ^28^ This Linac is designed for a “one‐step” patient setup that automatically applies couch shifts after an image‐guidance procedure.^22^ This eliminates the need for therapists to manually apply isocenter shifts in the room and will decrease overall treatment time.

We have recently installed a Halcyon V2.0 Linac in our institution and initial acceptance testing and commissioning data confirmed that the machine met manufacturer specifications as described above.^22^, ^23^, ^24^, ^25^, ^26^, ^27^, ^28^ We originally installed the Halcyon Linac for conventionally fractionated treatments but decided to commission it for extracranial SBRT treatments because of the superior image quality and performance capabilities aforementioned. To improve dose calculation accuracy, we have used the advanced AcurosXB algorithm^29^, ^30^, ^31^ to better account for heterogeneities including SBRT board and Halcyon couch insert. For mobile tumors, we account for tumor motion using both abdominal compression and 4D‐CT‐based target delineation. We performed an end‐to‐end test and independent validation test by delivering a SBRT prescription dose of 6.6 Gy to the MD Anderson's VMAT/IMRT credentialing phantom in treatment mode. All dosimetric criteria established by the IROC for SBRT treatments were satisfied.

A few early researchers have shown a fast and effective treatment delivery is possible using Halcyon Linac for conventionally fractionated breast, head, and neck, and prostate treatments with no reduction in plan quality when compared to other treatment modalities.^32^, ^33^, ^34^, ^35^ In a hypofractionated study, Knutson et al. reported a retrospective dosimetric analysis of intracranial stereotactic radiation therapy (SRT) treatments using the Halcyon Linac.^36^ In a 20‐patient study with a fractionation scheme of 30 Gy in five fractions, they demonstrated that acceptable plan quality for brain SRT is achievable using Halcyon coplanar geometry. Another recent study by Li et al. demonstrated that the Halcyon V2.0 can generate plan quality comparable to a SBRT‐dedicated C‐arm Linac for 6‐10 brain tumors (diameter >1.0 cm), using a single‐isocenter approach for intracranial radiosurgery.^37^ While these retrospective planning studies demonstrated acceptable plan quality, they did not use their plans for the patient treatment. In this report, we evaluate consecutive ten low‐ and intermediate‐risk prostate cancer patient's SBRT treatment plans who were recently treated on our Halcyon Linac. These plans were evaluated for plan quality, treatment delivery efficiency, and accuracy as part of commissioning and clinical implementation of our prostate SBRT program on this novel Linac. Moreover, this report provides the benchmark study for other clinics to begin prostate SBRT treatments on the Halcyon. This novel investigation is the first to focus on the evaluation and clinical implementation of clinically delivered prostate SBRT treatments on the Halcyon Linac.

## 2 MATERIALS AND METHODS

### 2.1 Patient population

After obtaining Institutional Review Board approval for our institute, ten consecutive localized low‐ and intermediate‐risk prostate cancer patients who underwent prostate SBRT treatments on our Halcyon Linac were selected for this study. All patients received 36.25 Gy in five treatments. These patients had a Gleason Scores of less than or equal to 7. All men included in this study were aged greater than 18 years old with a prostate cancer clinically staged T1c‐T2a. All patients were grade group 1‐3 prostate adenocarcinoma and had a PSA ≤ 10 ng/Ml. Exclusion criterion included androgen deprivation therapy, prior prostate surgery, prior pelvic radiation therapy, crohn's diseases, ulcerative colitis and international prostate symptom score (IPSS) ≥15. All patients had no evidence of enlarged lymph nodes seen on CT/MRI images and bone scans revealed no evidence of distance bony metastases.

### 2.2 Imaging and target delineation

All patients were immobilized using the Body Pro‐Lok^TM^ platform (CIVCO system, Orange City, IA) in the supine position with their arms up. A Knee cushion was used to immobilize the knees. Patients were instructed to present for CT simulation and treatment with a comfortably full bladder and empty rectum. To ensure a relatively empty rectum, patients were instructed to use metamucil beginning 3‐day prior to simulation and daily throughout the course of treatment. A free‐breathing planning 3D‐CT scan was acquired on a GE Lightspeed 16 slice CT scanner (General Electric Medical Systems, Waukesha, WI) with 512 × 512 pixels at 2.5‐mm slice thickness in the axial helical mode. The planning 3D‐CT images were imported into Eclipse treatment planning system (TPS, Version 15.6, Varian Medical Systems, Palo Alto, CA) for contouring the entire prostate capsule as a clinical target volume (CTV) and the OAR. The planning target volume (PTV) was generated by adding a 3 mm symmetric margin around the prostate capsule per RTOG‐0938 recommendation.^12^ The average PTV size was 117.0 ± 34.0 cc (range, 73.0 to 186.0 cc), corresponding to an average PTV diameter of 6.0 ± 0.6 cm (range, 5.2 to 7.1 cm). OAR contours included rectum, bladder, penile bulb, femoral heads, skin, and urethra per RTOG‐0938 requirement. In addition, small bowel and sigmoid were also contoured for dose reporting.

### 2.3 Halcyon VMAT plans

All clinical VMAT prostate SBRT plans were generated in the Eclipse TPS using two‐full arcs on a Halcyon Linac (Varian Medical Systems, Palo Alto, CA) with a 6MV‐FFF (800 MU/min) beam. The isocenter position was set to the geometric center of the PTV and arcs had collimator angles of 30° and 330° that were chosen to reduce the MLC tongue‐and‐groove leakage dose to normal tissue. A dose of 36.25 Gy in five fractions was prescribed per RTOG 0938 protocol compliance and was normalized to ensure at least 95% of the PTV received 36.25 Gy. No hotspots to the PTV greater than 107% were allowed. All clinical treatment plans were optimized with the photon optimizer (PO) MLC algorithm and the final dose calculation was performed with an advanced Acuros‐based (Varian Eclipse TPS, Version 15.6) dose calculation algorithm^29^, ^30^, ^31^ on the planning 3D‐CT images with a 1.25 mm calculation grid size (CGS). The Halcyon couch and SBRT board were included in the final dose calculation. Dose to medium reporting mode was enabled and the planning objectives followed the RTOG‐0938 requirements for prescription isodose surface coverage, target dose homogeneity, high and low dose spillages and dose to limiting OAR.^12^ These patients were treated every other day using a Halcyon kV‐CBCT imaging protocol.

### 2.4 Truebeam VMAT plans

For comparison, all patients’ plans were retrospectively reoptimized in the Eclipse TPS using the same numbers of full arcs, identical collimator rotations, and arc geometry on our SBRT‐dedicated C‐arm Truebeam Linac (Varian Medical Systems, Palo Alto, CA) equipped with a standard millennium 120 MLC. A nominal 6MV‐FFF beam was selected with a maximal achievable dose rate setting of 1400 MU/min. Additionally, the Halcyon couch was removed and the Truebeam couch was inserted into the plan. Optimization objectives were identical per Halcyon VMAT plans. Identical dose calculation algorithm, CGS, dose reporting, convergence mode, and PO MLC optimizer were used for the Truebeam VMAT prostate SBRT plans. In contrast to the jawless Halcyon VMAT plans, Truebeam VMAT plans enabled jaw‐tracking option during plan optimization to further minimize the out‐of‐field dose. All Truebeam VMAT plans’ PTV coverage was normalized identically to clinical Halcyon VMAT plans with no hotspots higher than the respective clinically treated plans.

### 2.5 Plan comparison

The clinical Halcyon VMAT and Truebeam VMAT plans were compared via the RTOG‐0938 prostate SBRT protocol (Arm 1) for target coverage and dose to OAR. Both plans were evaluated for dose to the rectum (1 cc, 3 cc, 90%, 80%, and 50% of rectum), bladder (1 cc, 90% and 50% of bladder), penial bulb (maximum and 3 cc), femoral heads (10 cc), skin (maximum), and urethra (maximum). Additionally, target conformity index (CI) defined as the ratio of prescription isodose volume to the PTV and the maximum dose at any point 2 cm away from the PTV margin in any direction (D2cm) were recorded. Treatment delivery efficiency and accuracy was documented by recording the total number of monitor units (MU) per fraction; the ratio of total number of MU per fraction to the prescription dose in cGy defined as the modulation factor (MF) and beam‐on time (BOT) was recorded during the delivery of the quality assurance (QA) plan at each respective linac. Dosimetric verification of both plans was performed using the portal dosimetry (PD) measurement QA procedure.^38^, ^39^, ^40^ A gamma evaluation criteria of 2%/2mm with a low dose threshold set to 5% were used. The electronic portal imaging device (EPID, aS1200 flat panel detector, Varian Medical Systems, Palo Alto, CA) mounted on the Truebeam and Halcyon Linacs were used for measurement. This detector has an active area of 400 mm × 400 mm with a high‐resolution pixel size of 0.34 mm at a 150 cm nominal source to imager distance. The mean and standard deviation for each dose metric was compared using a two‐tailed paired student's t‐test (p value of < .05 statistically significant) for all dosimetric parameters, target coverage, OAR doses, and treatment delivery parameters.

## 3 RESULTS

### 3.1 Target coverage, CI and D2cm

Table 1 summarizes the target coverage including both high and intermediate dose‐spillage indices for both plans. All plans were acceptable per RTOG‐0938 requirements. Clinical Halcyon VMAT plans showed no statistical difference in target coverage and conformity with respect to Truebeam VMAT plans. The minimum dose to PTV was slightly better in the Halcyon VMAT plan for all patients for identical PTV D95 coverage of the both plans. The D2cm was statistically significant (*P* = 0.006) and consistently smaller for the Halcyon VMAT plans, indicating faster intermediate dose fall‐off around the prostate.

**TABLE 1:** Evaluation of target coverage and high and intermediate dose spillage for all ten prostate SBRT patients for both plans. Prescription was 36.25 Gy in 5 fractions.

	Parameters	Halcyon VMAT	Truebeam VMAT	RTOG‐0938 criteria (Gy)
Prostate, PTV	D_0.03 cc_ (Gy)	38.12 ± 0.33 (37.74–38.64)	38.10 ± 0.29 (37.60–38.50)	≤ 38.78
	D_95%_ (Gy)	36.25 ± 0	36.25 ± 0	≥ 36.25
	D_min_ (Gy)	34.83 ± 0.89 (34.35–35.79)	33.32 ± 1.18 (31.93–34.90)	≥ 34.4
High and intermediate dose spill	CI	1.00 ± 0.05 (0.97–1.12)	1.01 ± 0.05 (0.96–1.11)	—
	D2cm (%)	63.00 ± 5.19 (53.90–70.80)	65.79 ± 6.68 (54.90–75.90)	—

Mean ± SD (range) was reported.

### 3.2 Dose to OAR

The dosimetric differences (mean, standard deviation, and range) between clinical Halcyon VMAT and Truebeam VMAT plans for the OAR (rectum, bladder, penial bulb, femoral head, skin, and urethra) are listed in Table 2. No major and statistically significant dosimetric differences were observed. Both plans achieved RTOG‐0938 protocol compliance and were clinically acceptable for SBRT treatment. This suggests that dosimetrically equivalent and RTOG compliant prostate SBRT plans can be generated with the O‐ring Halcyon Linac compared to a SBRT‐dedicated Truebeam Linac. Doses to sigmoid and small bowel were small and similar between the plans and are not included in Table 2.

**TABLE 2:** Evaluation of dose to OAR for all ten prostate SBRT patients for both plans. Prescription was 36.25 Gy in five fractions.

Dose to OAR	Parameters (Gy)	Halcyon VMAT	Truebeam VMAT	RTOG‐0938 criteria (Gy)
Rectum	D_1cc_	32.25 ± 4.77 (25.38–36.65)	32.46 ± 4.55 (23.62–36.73)	≤ 38.06
	D_3cc_	29.29 ± 5.48 (22.03–34.33)	29.73 ± 4.86 (21.39–36.05)	≤ 34.40
	D_90%_	25.57 ± 4.77 (20.05–32.48)	26.05 ± 4.38 (21.28–32.54)	≤ 32.63
	D_80%_	21.13 ± 3.43 (16.92–26.93)	22.04 ± 3.49 (17.14–27.80)	≤ 29.00
	D_50%_	12.77 ± 4.26 (4.59–17.26)	13.39 ± 4.88 (3.83–18.18)	≤ 18.13
Bladder	D_1cc_	37.18 ± 0.24 (36.84–37.63)	37.28 ± 0.29 (36.81–37.79)	≤ 38.06
	D_90%_	22.67 ± 9.22 (3.02–31.48)	22.51 ± 9.44 (2.69–32.48)	≤ 32.63
	D_50%_	3.50 ± 2.64 (0.45–7.72)	3.29 ± 2.39 (0.39–7.91)	≤ 18.13
Penile bulb	D_0.03cc_	18.57 ± 15.45 (1.65–36.15)	17.91 ± 15.43 (1.69–36.21)	≤ 36.25
	D_3cc_	4.43 ± 4.03 (1.24–12.06)	3.98 ± 3.44 (1.27–11.19)	≤ 20.00
Femoral heads	D_10cc_	13.03 ± 1.65 (10.92–16.20)	12.83 ± 1.35 (10.58–15.45)	≤ 20.00
Skin	D_0.03cc_	11.45 ± 1.27 (9.25–12.97)	11.57 ± 0.75 (10.42–12.86)	≤ 30.00
Urethra	D_0.03cc_	37.26 ± 0.45 (36.63–37.89)	37.29 ± 0.42 (36.59–37.66)	≤ 38.78

Mean ± SD (range) was reported. None of the OAR parameters evaluated show statistically significant differences.

### 3.3 Treatment delivery efficiency and accuracy

Dose delivery efficiency was assessed by comparing the total number of MU and beam‐on time was estimated by recording portal dosimetry QA delivery time on both machines. Compared to clinical Halcyon VMAT plans, Truebeam VMAT plans delivered slightly higher total MU corresponding to higher beam modulation. Mean values of total MU and MF were 2562 and 3.53 for clinical Halcyon VMAT plans vs 2666 and 3.68 for the corresponding Truebeam VMAT plans (Table 3). Less MLC modulation with Halcyon is desirable as it may reduce MLC control point positioning errors. This highlights an added benefit of treating prostate SBRT on the Halcyon instead of a traditional C‐arm Linac. The beam‐on time and the PD QA pass rates for the clinical Halcyon VMAT vs Truebeam VMAT plans are shown in Table 3. Despite the less total MU and small MF, the mean total beam‐on time at Halcyon Linac plans (3.2 minutes, up to 3.8 minutes) was slightly longer compared to Truebeam VMAT plans (2.1 minutes, up to 2.5 minutes) (*P* < 0.001). However, one can argue this comparison is unfair as the maximal achievable dose rate on the Halcyon is 800 MU/min vs 1400 MU/min on Truebeam. While the beam‐on time is increased, overall treatment time will be similar because the Halcyon's one‐step patient setup, faster 15‐second kV‐CBCT imaging and auto matching procedure compared to Truebeam. We have observed that this actually additionally lower the total time a patient is on the Halcyon treatment couch and potentially minimize errors due to intrafraction prostate motion.

**TABLE 3:** Comparison of average values of treatment delivery parameters (and range) between the clinical Halcyon VMAT and replanned Truebeam VMAT plans for all ten prostate SBRT patients.

Beam delivery parameters	Halcyon VMAT	Truebeam VMAT	*P*‐value
Total monitor units (MU)	2562 ± 368 (1929–3029)	2666 ± 259 (2425–3007)	*P = 0.525*
Modulation factor (MF)	3.53 ± 0.51 (2.66–4.18)	3.68 ± 0.6 (3.34–4.15)	*P = 0.525*
BOT (min)	3.20 ± 0.46 (2.41–3.79)	2.1 ± 0.39 (1.93–2.45)	***P < 0.001***
Treatment time (min)	8.20 ± 0.46 (7.41–8.79)	9.90 ± 0.19 (9.73–10.15)	***P < 0.001***
Pretreatment PD QA, γ‐pass rate (%) [2%/2mm]	98.6 ± 1.5 (95.8–100.0)	98.3 ± 2.0 (98.3–100.0)	*P = 0.730*

Statistically significant values are highlighted in bold.

Treatment delivery accuracy of the prostate SBRT plans was evaluated by delivering each plan in QA measurement to both Linacs via on‐board EPID imager and recording the gamma analysis pass rates via portal dosimetry. Our clinic's SBRT QA program complies with the TG‐218 recommended pretreatment QA tolerance level (overall pass rate ≥95% at a γ– criteria of 2%/2mm with low threshold of 5%).^40^ All plans satisfied this requirement and were similar. The dose delivery accuracy of the Halcyon VMAT and the corresponding Truebeam VMAT plans were 98.6 ± 1.5% (range 95.8–100.0%) and 98.3 ± 2.0% (range 95.3–100%), respectively.

### 3.4 Example patient treatment

**FIG. 1:**
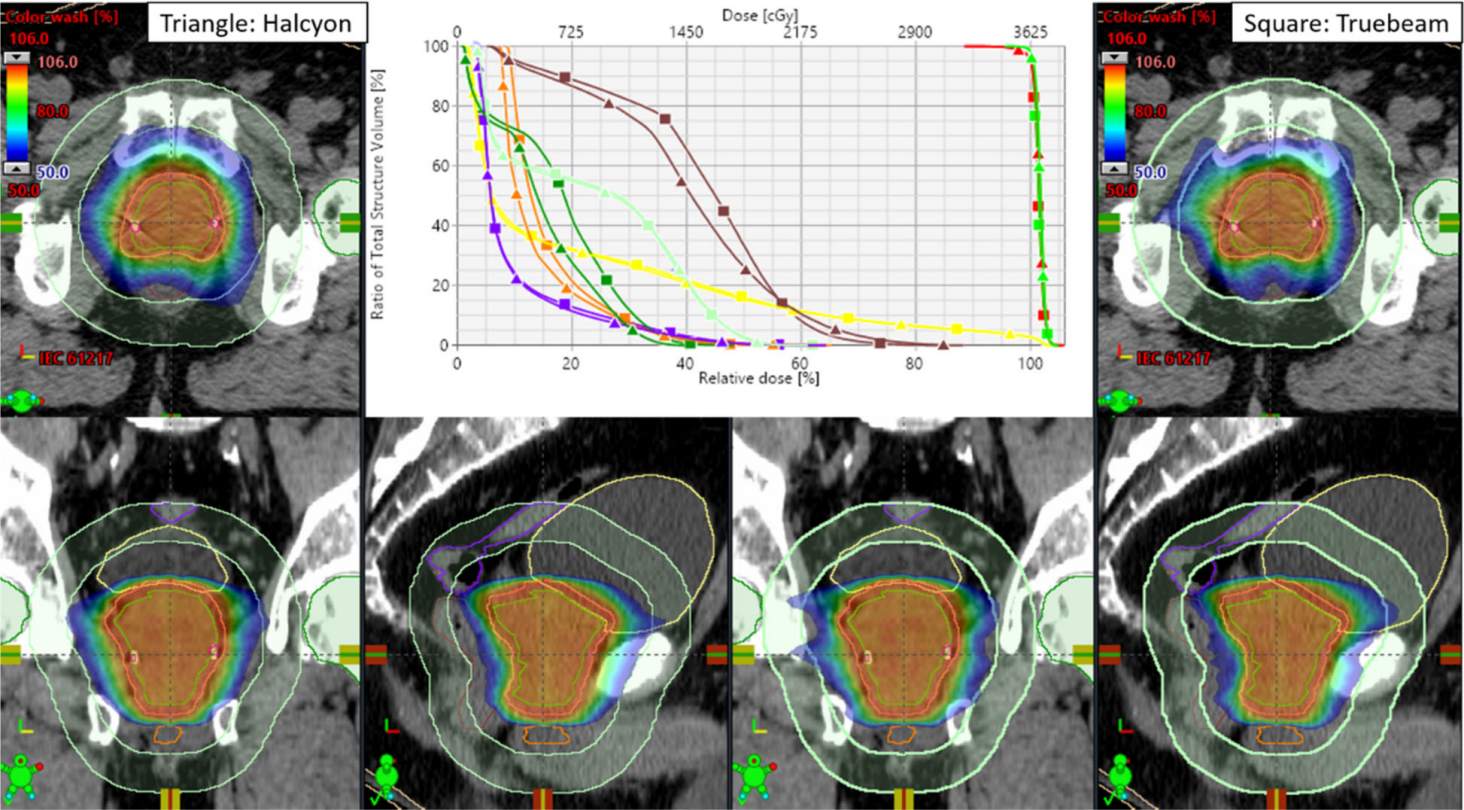
The SBRT isodose colorwash through the isocenter location for the clinical Halcyon VMAT (left panel) vs Truebeam VMAT plan (right panel) is shown for an example patient. A few critical structures shown are rectum (brown), bladder (yellow), sigmoid (purple), femoral heads (dark green), penial bulb (orange). The D2cm (light green) ring is shown. The top middle panel shows the DVH comparison for both plans with the identical PTV coverage (red); 95% of the PTV received 100% of the prescription dose and similar dose (~100%) to the prostate capsule (green). Triangles are clinical Halcyon VMAT and squares are the corresponding Truebeam VMAT plan. Identical target coverage and similar OAR sparing were achieved on both plans.

Fig. 1 shows the SBRT dose distribution in the axial, coronal and sagittal views through the isocenter plane (cross hair) for an example prostate SBRT patient #2. The Halcyon VMAT (left panel) and the corresponding Truebeam VMAT (right panel) plan is shown. Halcyon VMAT produced a similar or tighter 50% isodose distribution (see blue isodose line with respect to D2cm ring) compared to the Truebeam VMAT plan. The DVH (top middle panel) for targets (prostate capsule and PTV) and OAR suggest the Halcyon provides dosimetrically comparable plans with slightly better (but clinically insignificant) normal tissue sparing. In this case, the PTV size was 126.6 cc (6.2 cm diameter). The CI and D_2cm_ slightly favored the Halcyon plans and were 1.05 vs 1.06 and 61.5% vs 64.0% for clinical Halcyon VMAT vs Truebeam VMAT plan, respectively slightly favoring Halcyon plan. All dosimetric parameters (including dose to OAR) were similar between the plans and within the RTOG‐0938 compliance criteria.

**FIG. 2:**
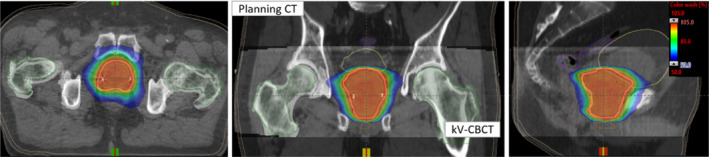
Axial, coronal and sagittal views of Halcyon kV‐CBCT images (see inset) co‐registered with planning CT images (see back of coronal and sagittal views) used for image guided prostate SBRT treatment on Halcyon. In addition to anatomical landmarks, the planned dose cloud was overlaid for this treatment. Halcyon kV‐CBCT images were acquired in the treatment position in free breathing and rigid‐registration was performed automatic image‐registration followed by manually fine‐tuning the fiducial markers for finer alignment.

Fig. 2 shows the patient setup kV‐CBCT images of the same example patient previously shown in Fig. 1. The planned isodose color wash is superimposed with the daily Halcyon kV‐CBCT images after the three degrees‐of‐freedom (3DoF) translational couch corrections were applied. This patient was initially positioned using external marks and in‐room lasers, followed by a one‐step setup and a 15‐second pretreatment iterative kV‐CBCT scan. Halcyon kV‐CBCT pelvis imaging protocol parameters (125 kV, 1080 mAs) were used with 512 × 512 pixels and 2.0 mm slice thickness. An in‐house SBRT/IGRT protocol was applied to coregister the pretreatment kV‐CBCT with the planning CT scans (see Fig. 2). Image registration was performed automatically based on region of interest and bony landmarks. Registration was followed by a manual refinement of the implanted fiducial markers matching and confirmed by the treating physician and physicist. The patient position was then corrected for 3DoF with respect to the isocenter according to the location of implanted fiducial markers and the treatment was delivered. Our departmental SBRT protocol limits all translational 3DoF couch corrections to less than ±3.0 mm in each direction for all SBRT treatments.

## 4 DISCUSSION

In this report, we have presented the plan quality, treatment delivery efficacy and accuracy of prostate SBRT treatments using the O‐ring Halcyon Linac. We found that all VMAT prostate SBRT plans generated using Halcyon Linac had similar or better dosimetric plan quality compared to SBRT‐dedicated Truebeam VMAT plans. This included: target conformity, tumor dose homogeneity, and intermediate dose fall‐off around the prostate. Additionally, all highly conformal clinical Halcyon VMAT plans met RTOG‐0938 requirements and achieved similar target coverage (see Table 1) compared to Truebeam VMAT plans. All Halcyon VMAT plans provided similar OAR (rectum, bladder, penial bulb, femoral heads, skin, and urethra) sparing and met SBRT protocol requirements (see Table 2). It should be noted that Halcyon VMAT plans required less MU to deliver the same prescribed dose. Although the beam‐on times were longer for Halcyon plans (about 1.1 minutes, on average) than Truebeam VMAT plans, overall treatment times are similar between the Halcyon and traditional C‐arm Linacs. As previously stated, the Halcyon Linac has a lower maximum available dose rate setting (800 MU/min) than Truebeam (1400 MU/min) but can compensate for this via one‐step patient setup via automatic applied shifts and faster image‐guided procedure. Similar pass rates of PD QA measurements suggest that similar delivery accuracy can be achieved between the plans.

Several prospective trials of prostate SBRT treatment of low‐ and intermediate‐risk prostate cancer have shown impressive patient outcomes with the robotic CyberKnife radiosurgery or helical tomotherapy units as described above.^1^, ^2^, ^3^, ^4^, ^5^, ^6^, ^7^, ^8^, ^9^ These outcomes have recently been compared using a SBRT‐dedicated C‐arm Linac such as Truebeam with FFF‐beam and VMAT planning technique.^19^, ^20^, ^21^ For instance, D'Agostino and colleagues recently published the results of their phase II trial evaluating the efficacy and toxicity of low‐ and intermediate‐risk prostate SBRT patients treated on Truebeam Linac via one to two full arcs VMAT plans for 35 Gy in five fractions, treated every other day with the average treatment times of 15 minutes.^21^ At the median follow‐up of 27 months, only two intermediate risk of 90 patients experienced a biochemical failure.^21^ They additionally reported patient's in this cohort experienced mild toxicity profiles with no reduced quality of life.^21^ Similar to their study, utilizing two‐full VMAT arcs our Halcyon Linac can deliver prostate SBRT treatment in less than 10 minutes compared to traditional robotic CyberKnife (~40 minutes) or Tomotherapy unit (20‐30 minutes).^1^, ^2^, ^3^, ^4^, ^5^, ^6^, ^7^, ^8^, ^9^, ^10^ By significantly shortening the overall treatment time, the risk of deviating from planned dose distribution could be decreased as the patient is less likely to move from the treatment position. This could minimize and potentially minimize intrafraction motion errors. Our Halcyon VMAT plans showed similar dose to target and dose to the entire OAR in (including rectum) compared to the SBRT‐dedicated Truebeam VMAT plans. Halcyon VMAT plans met all the requirements set forth by RTOG‐0938 compliance criteria, therefore we do not expect any major acute or late toxicity related to the prostate SBRT treatment. However, clinical follow‐up results of tumor local‐control and treatment‐related toxicities are necessary and is ongoing to confirm patient outcomes.

One drawback to treating prostate SBRT patients on the Halcyon Linac is there is currently no way to apply 6DoF couch corrections. Only 3DoF translational corrections can be made. Our Truebeam Linac is capable of applying 6DoF couch corrections and may better reduce the residual rotational setup errors in the treatment of prostate SBRT. However, until now, we do not know the exact dosimetric differences of 6DoF couch corrections for prostate SBRT treatment on Halcyon and this is subject of our ongoing research. The second constraint is the Halcyon's highest available maximal dose‐rate setting of 800 MU/min. While the Halcyon has a similar beam profile to the Truebeam Linac^23^ this is significantly affecting the beam‐on time compared to the Truebeam's VMAT plans (1400 MU/min). For all Truebeam VMAT plans, the dose rate of 1400MU/min was achieved on each arc for this prescription. Therefore, achieving maximal dose rates of greater than 800MU/min on Halcyon could potentially succeed overall Truebeam's treatment time and improve treatment delivery efficiency of prostate SBRT.

In summary, each Halcyon prostate SBRT plan was rigorously evaluated using the dosimetric compliance criteria set forth by RTOG‐0938 compliance requirements including relevant treatment delivery parameters listed in Tables 1, 2, 3. All parameters were deemed acceptable for treating prostate SBRT patients on Halcyon with overall faster treatment time of less than 10 minutes, potentially benefiting patients who cannot lie flat in the treatment position for longer treatment time, minimizing intrafraction motion error and improving clinic efficiency. Based on this research and other previous studies, we are planning to expand our Halcyon Linac to treat other disease sites such as fractionated stereotactic treatment of brain tumors^36^, ^37^ any abdominal and pelvic nodes SBRT and selected liver, pancreas or adrenal glands SBRT patients. Due to lower total MU per treatment and relatively smaller beam‐on time, deep inspiration breath‐hold to liver or pancreatic SBRT treatments on Halcyon Linac warrant future investigation. Moreover, our future research includes quantifying the dosimetric effects of 6DoF rotational couch corrections errors for prostate SBRT treatments and developing a new knowledge‐based planning model for the prospective prostate SBRT treatments on Halcyon Linac.

## 5 CONCLUSION

In this report, we have demonstrated the treatment planning feasibility, delivery efficiency and accuracy, and clinical implementation of prostate SBRT treatments on the O‐ring Halcyon Linac for selected low‐and intermediate‐risk prostate cancer patients. The results of this study indicate the treatment of prostate SBRT patients on the Halcyon Linac is capable of a safe, effective, accurate, and clinically comparable to SBRT‐dedicated Truebeam Linac. This demonstrates the potential of a mainstream fast‐rotating O‐ring Halcyon Linac for prostate SBRT patients. For clinics only equipped with a Halcyon Linac, we recommend they commission and begin treating prostate SBRT patients, if available. This could better provide access to standardized, fast, and curative SBRT treatment for the large and underserved cohort of prostate cancer patients. Clinical outcomes of tumor local‐control rates and toxicity profiles of prostate SBRT patients treated on Halcyon Linac are warranted.

## AUTHOR's CONTRIBUTIONS

DP conceived the project. DP, TT, JS, and JV collected data, performed measurement, and analyzed the data. FAZ, AMG, SES, and WSC provided clinical expertise and supervision of the paper. DP drafted the manuscript and all coauthors reviewed, revised, and approved the final manuscript.

## CONFLICT OF INTEREST

None.

## References

[1] Choi C , Cho G , Kim K , et al. Stereotactic radiation therapy of localized prostate cancer using CyberKnife. Int J Radiat Oncol Biol Phys. 2007;69:S375.

[2] Katz A , Santoro M , Ashley R , et al. Stereotactic body radiotherapy for organ‐confined prostate cancer. BMC Urol. 2010;10:1. 20122161 10.1186/1471-2490-10-1 PMC2831888

[3] Freeman E , King R . Stereotactic body radiotherapy for low‐risk prostate cancer: five‐year outcomes. Radiat Oncol. 2011;6:3. 21219625 10.1186/1748-717X-6-3 PMC3022740

[4] Kang J , Cho C , Choi C , et al. Image‐guided stereotactic body radiation therapy for localized prostate cancer. Tumori. 2011;97:43‐48. 21528663 10.1177/030089161109700109

[5] Boike T , Lotan Y , Cho L , et al. Phase I dose‐escalation study of stereotactic body radiation therapy for low‐ and intermediate‐risk prostate cancer. J Clin Oncol. 2011;29:2020‐2026. 21464418 10.1200/JCO.2010.31.4377 PMC3138546

[6] McBride S , Wong D , Dombrowski J , et al. Hypofractionated stereotactic body radiotherapy in low‐risk prostate adenocarcinoma: Preliminary results of a multi‐institutional phase 1 feasibility trial. Cancer. 2012;118:3681‐3690. 22170628 10.1002/cncr.26699

[7] Yeoh E , Botten R , Butters J , et al. Hypofractionated versus conventionally fractionated radiotherapy for prostate carcinoma: final results of phase III randomized trial. Int J Radiat Oncol Biol Phys. 2011;81:1271‐1278. 20934277 10.1016/j.ijrobp.2010.07.1984

[8] Pollack A , Buyounouski M , Horwitz E , et al. Five year results of a randomized external beam radiotherapy hypofractionation trial for prostate cancer. Int J Radiat Oncol Biol Phys. 2011;81(suppl):S1.

[9] King C , Brooks J , Gill H , et al. Long‐term outcomes from a prospective trial of stereotactic body radiotherapy for low‐risk prostate cancer. Int J Radiat Oncol Biol Phys. 2012;82:877‐882. 21300474 10.1016/j.ijrobp.2010.11.054

[10] Macias V , Blanco M , Perez‐Romasanta L . Initial experience with stereotactic body radiation therapy for localized prostate cancer using helical tomotherapy. Clin Transl Oncol. 2014;16:380‐385. 23928833 10.1007/s12094-013-1089-y

[11] Scobioala S , Kittel C , Elsayad K , et al. A treatment planning study comparing IMRT techniques and cyber knife for stereotactic body radiotherapy of low‐risk prostate carcinoma. Radiation Oncology. 2019;14:143. 31399115 10.1186/s13014-019-1353-6 PMC6689170

[12] NRG RTOG‐0938 Protocol . A randomized phase II trial of hypofractionated radiotherapy for favorable risk prostate cancer, (2016); 1‐66.

[13] Benedict S , Yenice K , Followill D , et al. Stereotactic body radiation therapy: The report of AAPM Task Group 101. Med Phys. 2010;37:4078‐4100. 20879569 10.1118/1.3438081

[14] Arcangeli S , Scorsetti M , Alongi F . Will SBRT replace conventional radiotherapy in patients with low‐intermediate risk prostate cancer? A review. Crit Rev Oncol Hema. 2012;84:101‐108. 10.1016/j.critrevonc.2011.11.009 22257653

[15] Lin Y , Lin K , Ho H , et al. Treatment plan comparison between stereotactic body radiation therapy techniques for prostate cancer: Non‐isocentric CyberKnife versus isocentric RapidArc. Physica Med. 2014;30:654‐661. 10.1016/j.ejmp.2014.03.008 24726212

[16] MacDougall N , Dean C , Muirhead R . Stereotactic body radiotherapy in prostate cancer: is rapidarc a better solution than cyberknife? Clin Oncol (R Coll Radiol). 2014;26:4‐9. 24071450 10.1016/j.clon.2013.08.008

[17] Scorsetti M , Alongi F , Castiglioni S , et al. Feasibility and early clinical assessment of flattening filter free (FFF) based stereotactic body radiotherapy (SBRT) treatments. Radiat Oncol. 2011;6:113. 21910868 10.1186/1748-717X-6-113 PMC3179946

[18] Xiao Y , Kry S , Popple R , et al. Flattening filter‐free accelerators: a report from the AAPM therapy emerging technology assessment work group. J Appl Clin Med Phys. 2015;16:12. 10.1120/jacmp.v16i3.5219 PMC5690108 26103482

[19] Alongi F , Cozzi L , Argangeli S , et al. Linac based SBRT for prostate cancer in 5 fractions with VMAT and flattening filter free beams: preliminary report of phase II study. Radiat Oncol. 2013;8:171. 23835141 10.1186/1748-717X-8-171 PMC3718706

[20] Alongi F , Mazzola R , Fiorentino A . Phase II study of accelerated Linac‐based SBRT in five consecutive fractions for localized prostate cancer. Strahlenther Onkol. 2019;195:113‐120. 30003292 10.1007/s00066-018-1338-7

[21] D'Agostino G , Franzese C , Rose F , et al. High‐quality Linac‐based stereotactic body radiation therapy with flattening filter free beams and VMAT for low‐intermediate risk prostate cancer. A mono‐institutional experience with 90 patients. Clin Oncol (R Coll Radiol). 2016;28:e173‐e178. 27389021 10.1016/j.clon.2016.06.013

[22] Varian Halcyon Linac, Customer User Manual. Palo Alto, CA: Varian Medical Systems; 2019.

[23] Fogliata A , Cayez R , Garcia R , et al. Technical note: flattening filter free beam from Halcyon linac: evaluation of the profile parameters for quality assurance. Med Phys. 2020. 10.1002/mp.14217 32367534

[24] Gao S , Netherton T , Chetvertkov A , et al. Acceptance and verification of the Halcyon‐Eclipse linear accelerator‐treatment planning system without 3D water scanning system. J Appl Clin Med Phys. 2019;20:111‐117. 10.1002/acm2.12719 PMC6806699 31553525

[25] Gay S , Netherton J , Cardenas E , et al. Dosimetric impact and detectability of multi‐leaf collimator positioning errors on Varian Halcyon. J Appl Clin Med Phys. 2019;20:47‐55. 31294923 10.1002/acm2.12677 PMC6698762

[26] Lim Y , Dragojevi'c I , Hoffman D , et al. Characterization of the HalcyonTM multileaf collimator system. J Appl Clin Med Phys. 2019;20:106‐114. 30889312 10.1002/acm2.12568 PMC6448159

[27] Jarema T , Aland T . Using the iterative kV CBCT reconstruction on the Varian Halcyon linear accelerator for radiation therapy planning for pelvis patients. Phys Med. 2019;6(8):112‐116. 10.1016/j.ejmp.2019.11.015 31783220

[28] Cai B , Laugeman E , Mazur T , et al. Characterization of a prototype rapid kilovoltage x‐ray image guidance system designed for a ring shape radiation therapy unit. Med Phys. 2019;46:1355‐1370. 30675902 10.1002/mp.13396 PMC8188470

[29] Eclipse Algorithms Reference Guide (Version 15.6). Palo Alto, CA: Varian Medical Systems; 2019.

[30] Vassiliev O , Wareing T , McGhee J , et al. Validation of a new grid‐based Boltzmann equation solver for dose calculation in radiotherapy with photon beams. Phys Med Biol. 2010;55:581‐598. 20057008 10.1088/0031-9155/55/3/002

[31] Kroon P , Hol S , Essers M , et al. Dosimetric accuracy and clinical quality of Acuros XB and AAA dose calculation algorithm for stereotactic and conventional lung volumetric modulated arc therapy plans. Radiat. Oncol. 2013;8:149. 23800024 10.1186/1748-717X-8-149 PMC3723919

[32] Cozzi L , Fogliata A , Thompson S , et al. Critical appraisal of the treatment planning performance of volumetric modulated arc therapy by means of a dual layer stacked multileaf collimator for head and neck,breast, and prostate, technology in cancer research & treatment. 2019;17;I‐II. 10.1177/1533033818803882 PMC6176542 30295172

[33] O'Grady F , Barsky R , Anamalayil S , et al. Increase in superficial dose in whole‐breast irradiation with Halcyon straight‐through Linac compared with traditional C‐arm Linac with flattening filter: In vivo dosimetry and planning study. Adv Radiat Oncol. 2020;5:120‐126. 32051898 10.1016/j.adro.2019.07.011 PMC7004930

[34] Flores‐Martinez E , Kim Y , Yashar M , et al. Dosimetric study of the plan quality and dose to organs at risk on tangential breast treatments using the Halcyon linac. J Appl Clin Med Phys. 2019;20(7):58‐67. 31183967 10.1002/acm2.12655 PMC6612683

[35] Kim H , Huq M , Lalonde R , et al. Early clinical experience with Varian halcyon V2 linear accelerator: dual‐isocenter IMRT planning and delivery with portal dosimetry for gynecological cancer treatments. J Appl Clin Med Phys. 2019;20:111‐120. 10.1002/acm2.12747 PMC6839386 31660682

[36] Knutson N , Kennedy W , Reynoso F , et al. Intracranial stereotactic radiation therapy with a jawless ring gantry linear accelerator equipped with new dual layer multileaf collimator. Adv Radiat Oncol. 2020;5:482‐489. 32529144 10.1016/j.adro.2020.01.003 PMC7276691

[37] Li T , Irmen P , Liu H , et al. Dosimetric performance and planning/delivery efficiency of a dual‐layer stacked and staggered MLC on treating multiple small targets: A planning study based on single‐isocenter multi‐target stereotactic radiosurgery (SRS) to brain metastases. Front Oncol. 2019;9:7. 30723702 10.3389/fonc.2019.00007 PMC6349708

[38] Miri N , Keller P , Zwan B , Greer P . EPID‐based dosimetry to verify IMRT planar dose distribution for the aS1200 EPID and FFF beams. J Appl Clin Med Phys. 2016;6:292‐304. 10.1120/jacmp.v17i6.6336 PMC5690494 27929502

[39] Laugeman E , Heermann A , Hilliard J , et al. Comprehensive validation of halcyon 2.0 plans and the implementation of patient specific QA with multiple detector platforms. J Appl Clin Med Phys. 2020;21(7):39‐48. 10.1002/acm2.12881 PMC7386180 32368862

[40] Miften M , Olch A , Mihaildis D , et al. Tolerance limits and methodologies for IMRT measurement‐based verification QA: Recommendations of AAPM Task Group No. 218. Med Phys. 2018;45:e53‐e83. 29443390 10.1002/mp.12810

